# Polymorphisms in genes involved in estrogen and progesterone metabolism and mammographic density changes in women randomized to postmenopausal hormone therapy: results from a pilot study

**DOI:** 10.1186/bcr999

**Published:** 2005-02-23

**Authors:** Sarah J Lord, Wendy J Mack, David Van Den Berg, Malcolm C Pike, Sue A Ingles, Christopher A Haiman, Wei Wang, Yuri R Parisky, Howard N Hodis, Giske Ursin

**Affiliations:** 1 Department of Preventive Medicine, Keck School of Medicine, University of Southern California, Los Angeles, CA, USA; 2 Atherosclerosis Research Unit, Division of Cardiovascular Medicine, Keck School of Medicine, University of Southern California, Los Angeles, CA, USA; 3 Department of Radiology, Keck School of Medicine, University of Southern California, Los Angeles, CA, USA; 4 Department of Nutrition, University of Oslo, Norway

**Keywords:** clinical trial, estrogen and progestin therapy, genetic variants, mammographic density, randomized

## Abstract

**Introduction:**

Mammographic density is a strong independent risk factor for breast cancer, and can be modified by hormonal exposures. Identifying genetic variants that determine increases in mammographic density in hormone users may be important in understanding hormonal carcinogenesis of the breast.

**Methods:**

We obtained mammograms and DNA from 232 postmenopausal women aged 45 to 75 years who had participated in one of two randomized, double-blind clinical trials with estrogen therapy (104 women, taking 1 mg/day of micronized 17β-estradiol, E2), combined estrogen and progestin therapy (34 women, taking 17β-estradiol and 5 mg/day of medroxyprogesterone acetate for 12 days/month) or matching placebos (94 women). Mammographic percentage density (MPD) was measured on baseline and 12-month mammograms with a validated computer-assisted method. We evaluated polymorphisms in genes involved in estrogen metabolism (catechol-O-methyltransferase (*COMT *(Val158Met)), cytochrome P450 1B1 (*CYP1B1 *(Val432Leu)), UDP-glucuronosyltransferase 1A1 (*UGT1A1 *(<7/≥ 7 TA repeats))) and progesterone metabolism (aldo-keto reductase 1C4 (*AKR1C4 *(Leu311Val))) with changes in MPD.

**Results:**

The adjusted mean change in MPD was +4.6% in the estrogen therapy arm and +7.2% in the combined estrogen and progestin therapy arm, compared with +0.02% in the placebo arm (*P *= 0.0001). None of the genetic variants predicted mammographic density changes in women using estrogen therapy. Both the *AKR1C4 *and the *CYP1B1 *polymorphisms predicted mammographic density change in the combined estrogen and progestin therapy group (*P *< 0.05). In particular, the eight women carrying one or two low-activity *AKR1C4 *Val alleles showed a significantly greater increase in MPD (16.7% and 29.3%) than women homozygous for the Leu allele (4.0%).

**Conclusion:**

Although based on small numbers, these findings suggest that the magnitude of the increase in mammographic density in women using combined estrogen and progestin therapy may be greater in those with genetically determined lower activity of enzymes that metabolize estrogen and progesterone.

## Introduction

There is growing evidence that combined estrogen and progestin therapy (EPT) increases the risk of breast cancer more than estrogen therapy (ET) alone [1-6]. One important question is whether we can identify subgroups of women who are at a particularly greater risk of developing breast cancer if they use EPT or ET. Mammographic percentage density (MPD) is a strong independent breast cancer risk factor [7-10] and increases when women commence EPT. On average the change is 4 to 5% [11]; however, a sub-group of about 25% (range 10 to 40%) of women starting EPT undergo a substantial increase in MPD of at least 10% or an upgrade in the four-level Wolfe classification [12-17].

Although it is not known which factors modify change in MPD in ET or EPT users, it is important to identify such factors because they might also modify the increase in risk of breast cancer associated with ET or EPT. Data from the Postmenopausal Estrogen and Progestin Interventions trial showed that the increase in serum estrone is a strong predictor of MPD increase in women randomized to EPT [18], suggesting that factors affecting the absorption or metabolism of EPT are important. As a next step, we decided to investigate whether known or suspected functional variants in genes involved in hormone metabolism would predict changes in MPD in women randomized to ET and EPT. We selected genes whose products are known to modulate important aspects in estrogen metabolism such as catechol-O-methyltransferase (*COMT*), cytochrome P450 1B1 (*CYP1B1*) and UDP-glucuronosyltransferase 1A1 (*UGT1A1*)), or in progesterone metabolism (aldo-keto reductase 1C4 (*AKR1C4*)). As far as we know, this is the first study to investigate genetic determinants of MPD changes in women randomized to ET, EPT or placebo.

## Materials and methods

### Study subjects

Subjects were drawn from two randomized, double-blind, placebo-controlled studies [19,20], conducted by the Atherosclerosis Research Unit at the Keck School of Medicine of the University of Southern California.

The Estrogen in the Prevention of Atherosclerosis Trial (EPAT) [19] was a clinical trial conducted in postmenopausal women aged 45 years or older recruited from direct advertising. Eligible women had a serum estradiol level of less than 20 pg/ml and a fasting plasma low-density lipoprotein cholesterol of at least 130 mg/dl. Exclusion criteria were: use of postmenopausal hormone therapy for more than 10 years or within the previous month of the first screening visit; history of breast or gynecologic cancer; life-threatening disease with a prognosis of less than 5 years; fasting triglyceride level 400 mg/dl or more; high-density lipoprotein level less than 30 mg/dl; diastolic blood pressure more than 110 mmHg; current smoker; untreated thyroid disease; renal insufficiency (serum creatinine more than 2.5 mg/dl); fasting blood glucose more than 200 mg/dl. The 222 subjects enrolled in this study were randomized to receive either 1 mg/day of micronized 17β-estradiol (ET) or placebo over a period of 2 years.

The Women's Estrogen–Progestin Lipid-Lowering Hormone Atherosclerosis Regression Trial (WELL-HART) [20] was conducted in postmenopausal women aged 50 to 75 years with angiographically demonstrable coronary artery disease. Participants were recruited from five cardiac catheterization laboratories in Los Angeles County that serve patients with diverse backgrounds. Other criteria for inclusion and exclusion were as in EPAT except that smokers of fewer than 15 cigarettes a day were not excluded from participation in WELL-HART. A total of 226 subjects were randomized to receive either 1 mg/day of micronized 17β-estradiol with medroxyprogesterone acetate (MPA) at 5 mg/day for days 19 to 30 each month (EPT), ET or matching placebo over a period of 3 years [20].

In the present study, we included all subjects who had participated in EPAT or WELL-HART for a minimum of 12 months, who had a current US telephone number and a mammogram within the 18 months before randomization that was at least 2 months after any previous episodes of postmenopausal hormone use based on patient-reported date of cessation or, if unavailable, patient response to screening interview question about use of any hormone therapy (HT) use in the previous month, and who did not have breast implants or a history of breast cancer between randomization and the follow-up mammogram. Potentially eligible women who were willing to participate in this mammography density sub-study signed a written informed consent form and provided a blood sample or, if they lived outside the greater Los Angeles area, a buccal cell sample. The study protocol was approved by the Institutional Review Board at the University of Southern California.

Of the 222 subjects randomized in EPAT, 150 (68%) women were assessed as eligible for recruitment to the present study. The reasons why 72 women were not eligible for the current study were as follows: loss to follow-up (*n *= 27), death (*n *= 1), withdrawal from original trial (*n *= 42), breast implants (*n *= 1), and breast cancer diagnosed during the trial (*n *= 1). Of these 150 eligible women, we successfully contacted 149, and 146 (97%) consented and provided a blood (*n *= 131) or buccal (*n *= 15) specimen. An appropriately timed set of mammograms was available for 127 of these participants (85% of subjects contacted).

Of the 226 subjects randomized in WELL-HART, 163 (72%) were eligible for recruitment. The reasons why 63 women were not eligible for the current study were as follows: loss to follow-up (*n *= 16), death (*n *= 10), withdrawal from original trial (*n *= 34), breast cancer diagnosed during the trial (*n *= 1), and not competent to sign informed consent (*n *= 2). We successfully contacted 155 of the 163 eligible women; 140 (89%) of these consented and provided a blood (*n *= 134) or buccal (*n *= 6) specimen. Mammograms were available for 105 of these women (68% of subjects contacted).

Reasons for non-participation among eligible subjects were as follows: telephone contact unsuccessful (EPAT 1, WELL-HART 8), patient refusal (EPAT 3, WELL-HART 15), and specimen stored incorrectly (EPAT 1). Problems encountered in retrieving mammograms were as follows: baseline mammogram not eligible (EPAT 5, WELL-HART 25) and mammogram not located at the facility where it was taken and further tracking was unsuccessful (EPAT 10, WELL-HART 9). No follow-up mammogram was available for three EPAT subjects, and one WELL-HART subject was excluded because of technically poor mammographic images (see assessment score below).

### Data collection

#### DNA samples and genotyping

Genomic DNA was isolated from blood with a QIAamp 96 DNA Blood Kit (Qiagen, Valencia, CA). DNA isolation from buccal cells in mouthwash was performed with the Puregene DNA isolation method (Gentra Systems Inc., Minneapolis, MN). Genotyping for the following single nucleotide polymorphisms was performed using the fluorogenic 5'-nuclease assay (TaqMan Assay) [21]: *COMT *(Val158Met), *CYP1B1 *(Val432Leu), *UGT1A1 *(<7/≥ 7 TA repeats) and *AKR1C4 *(Leu311Val). No signal or an indeterminate signal was recorded for one or two specimens (less than 1%) in assays for each polymorphism; these results are recorded as missing and are excluded from our analysis of that gene. Of the 5% blinded quality-control repeats, each result matched that of the corresponding specimen in all assays except one for which one of the controls gave no signal.

#### Assessment of MPD change

A baseline mammogram performed closest to, but before, the subject's randomization date and a mammogram obtained 1 year after randomization were used to measure MPD change. The mean time between the two mammograms was 14.4 months (range 9 to 31), and this was similar in all the treatment groups. In EPAT the mean time was 13.5 months in the placebo group and in the ET group 14.4 months (*t*-test *P *= 0.27); in WELL-HART the mean time was 14.9 months in the placebo group, 15.4 months in the ET group and 14.5 months in the EPT group (analysis of variance *P *= 0.64).

All films were scanned at 150 dots per inch with a Cobrascan CX-812T scanner (Radiographic Digital Imaging Inc., Torrance, CA) with Adobe Image software. Mammographic density was determined with a computer-assisted validated method [22] that we have previously found strongly predicts breast cancer risk [10], and that we have shown identifies increases in MPD when women commence EPT [11]. One of us (GU) assessed all the mammograms for absolute density, and the breast area was measured by a research assistant trained by GU. MPD was calculated as the absolute dense area multiplied by 100 divided by the breast area. The correlation of repeated MPD readings performed on a subset of 14 mammograms at different times was 0.95. The density reader also assigned a 'difficulty assessment score' for each mammogram ranging from 1 to 6, which indicated how difficult it was to read the scanned image; a score of 1 was 'normal', and a score of 6 was 'impossible' for technical reasons. Mammograms from one WELL-HART patient were scored as 6, and this patient was excluded from the analysis. Scores of 4 or 5 were recorded for 31 of 233 patients (13.3%). This number did not vary significantly by study group, timing of mammogram (before versus after treatment) or treatment arm (*P *> 0.45 for all).

### Statistical analysis

We used general linear models to determine the least-square mean change in MPD for subjects in each treatment group overall and by genotype for each trial independently, and with the two trials combined. We adjusted for factors known or suspected to be associated with changes in mammographic density, namely race (White, African American, Latina, Asian/Pacific Islander), body mass index (BMI) at baseline (kg/m^2^), change in BMI on trial, age at baseline mammogram (years) and MPD at baseline and any past use of ET or EPT (ever/never). A multiplicative genotype × treatment interaction term was also included in the model to test for genotype differences in the treatment-related change in MPD. For our key findings we also report conservative adjustments of the significance levels by using the Bonferroni technique (36 comparisons, Bonferroni-adjusted α level = 0.0014).

The laboratory personnel and the mammographic density assessor were blinded to treatment and study assignment. The SAS statistical software package (SAS Institute Inc., Cary, NC) was used for all statistical analyses.

## Results

### Subject characteristics

The distribution of baseline characteristics for the 232 subjects included in this study was similar to that of the parent trials, except that the WELL-HART participants included in this study were more likely to have had an education beyond high school (52%) than the WELL-HART participants not included (32%; *P *= 0.0003). However, education level was not associated with mammographic density at baseline or change in density. Within each trial, baseline characteristics including age, parity, BMI, family history of breast cancer, education and genotype were similar across treatment groups, except that within WELL-HART the racial distribution differed significantly by treatment assignment (*P *= 0.01).

There were several differences between the two trials, reflecting the differences in the inclusion criteria and recruitment strategies. WELL-HART subjects were statistically significantly older, less educated, more obese, of higher parity, less likely to have used postmenopausal hormones and more racially diverse than EPAT subjects (Table 1). Three of these factors – age, parity and BMI – are known to be independently and inversely associated with mammographic density [7] and, as expected from these differences, the mean MPD at baseline was significantly lower in WELL-HART subjects than in EPAT subjects (WELL-HART 10.6%; EPAT 20.3%; *P *= 0.0001). However, change in MPD did not vary with MPD at baseline (Pearson correlation coefficient – 0.06, *P *= 0.33; Spearman correlation coefficient 0.03, *P *= 0.64).

The allelic frequencies for all genes were in Hardy–Weinberg equilibrium across both trials with the exception of *CYP1B1*, which showed statistically significant variation by ethnicity. Because Hardy–Weinberg equilibrium was maintained within each ethnic group, a systemic genotyping problem with this locus is unlikely.

### Change in MPD by treatment arm

On average, women assigned to placebo did not exhibit any change in MPD from baseline (Table 2). Women assigned to ET in each trial showed a similar 4 to 5% increase in MPD over placebo in each trial (EPAT *P *= 0.0001, WELL-HART *P *= 0.02). Women assigned to EPT in WELL-HART exhibited the greatest mean change in MPD (7.8%); this was statistically significantly greater than placebo (*P *= 0.0005) but not significantly different from the ET groups (*P *= 0.32). To determine whether this was due to a change in breast area or in the amount of dense tissue in the breast (absolute density), we examined the effect of treatment on changes in dense area and changes in the total breast area. The treatment effect was observed when the analysis was undertaken for change in mammographic absolute density. The adjusted mean change in absolute density was greatest in women assigned to EPT (10.5 cm^2^) in comparison with women assigned to placebo (- 0.27 cm^2^, *P *= 0.005) but not significantly different from women assigned to ET (9.4 cm^2^, *P *= 0.76). No treatment effect was observed on change in breast area (*P *= 0.31). The remaining analyses are therefore restricted to changes in MPD.

Overall, one subject (1%) assigned to placebo showed a 10% increase in MPD over 12 months, compared with 17% of subjects assigned to ET and 32% assigned to EPT. Increases in MPD in the EPT arm were apparent within each ethnic group.

### Genetic determinants of MPD change

Because there was no statistical significant heterogeneity in the ET effect between the two studies (MPD change 4.0% and 5.6% respectively, *P *for homogeneity of effect = 0.76), we combined the results of both studies to provide more power to investigate a treatment interaction between genotype and change in MPD. There was no statistically significant association between genotype and baseline MPD in either trial or in the trials combined (data not shown). Overall there was also no evidence for an association between change in MPD and genotype in women randomized to ET (Table 3). However, in EPAT there was a statistically significant increase in MPD in women in the ET arm who possessed the *COMT *Met/Met genotype compared with those with the Val/Val genotype (*P *= 0.02, *P *for ET–genotype interaction = 0.17), but no such association was observed in the WELL-HART ET arm.

Two of the genes studied modified the MPD changes associated with EPT use. Both the Val/Val and Leu/Val genotypes of the *AKR1C4 *gene were associated with a statistically significant increase in density compared with the Leu/Leu genotype among women assigned to EPT (*P *= 0.0001, 0.0007, respectively; *P *= 0.004, 0.03, respectively, corrected for 36 multiple comparisons). However, there were only seven women heterozygous and one woman homozygous for the Val allele. The *AKR1C4*–treatment interaction was statistically significant (*P *= 0.001). When analyses were restricted to the WELL-HART study, the probabilities for the Val/Val and Leu/Val genotypes in comparison with the Leu/Leu genotypes were *P *= 0.001, 0.003, respectively, and the treatment interaction *P *= 0.05).

There was also a statistically significant association between the *CYP1B1 *genotype and MPD change among women taking EPT. The Leu/Leu genotype of *CYP1B1 *was associated with a statistically significantly greater MPD change in women assigned to EPT than the Val/Val genotype (*P *= 0.03, not significant after correction for multiple comparisons). However, heterozygotes for this polymorphism showed the smallest increase in MPD. The interaction between ET/EPT and *CYP1B1 *was statistically significant (*P *= 0.0004).

The results were similar when analyses were restricted to the WELL-HART study; the probability for the Leu/Leu genotype was *P *= 0.09 and treatment interaction *P *= 0.006.

The results for the *COMT *gene and the *UGT1A1 *genes were also similar when analyses were restricted to the WELL-HART study (results not shown).

## Discussion

In this study we found that women randomized to ET and EPT had a statistically significant mean increase in MPD over 12 months compared with women assigned to placebo, with the women assigned to EPT having the greatest mean increase in MPD. These findings are consistent with those (using the same reader and same method) from the only published placebo-controlled randomized clinical trial with EPT [11]. Interestingly, there was a similar effect of ET in two diverse study populations in the current study despite significant differences in MPD at baseline and other potential confounders (age, BMI and parity) between these two populations. The lack of a statistically significant difference between the increase in MPD in the EPT arm and the ET arms might have been due to a small EPT sample size of 34 subjects.

In data from the Postmenopausal Estrogen and Progestin Interventions (PEPI) trial, a greater increase in serum estrone level as a function of treatment was a significant predictor of MPD increase in women randomized to EPT, but not in women randomized to ET alone [18]. The PEPI study had no data on how serum progesterone or progestin levels changed. However, these results raised the possibility that factors associated with hormone absorption or metabolism are important determinants in how the breast tissue reacts to EPT.

Medroxyprogesterone acetate has a similar structure and metabolic pathway to progesterone. After medroxyprogesterone is ingested it undergoes reduction and hydroxylation in the small intestine [23,24]. After absorption it undergoes further metabolism in the liver, including 3α-hydroxylation (*AKR1C4*) [25]. The Leu311Val polymorphism on *AKR1C4 *has been associated with a 66 to 80% decrease in the catalytic activity of the enzyme [26]. Consistent with this was our finding that subjects randomized to EPT who were heterozygotes or homozygotes for this low-activity allele showed significantly greater increases in MPD than homozygotes for the wild-type Leu/Leu allele. In addition, the one subject possessing two copies of the Val allele showed the greatest increase in MPD, suggesting a potential allelic dosage effect.

A number of studies have investigated the role of estrogen metabolism on breast cancer risk. The major forms of estrogen, namely estrone and estradiol, are hydroxylated into 2-, 4- or 16-hydroxyestrogens. Initially, much research focused on the role of 2- and 16-hydroxy metabolites, with most of the later studies finding no protective effects of a high 2- to 16α-hydroxyestrone ratio [27-32]. In contrast, newer research suggests that the important question is how much estrogen is metabolized down the 4-hydroxy pathway, because the 4-hydroxy products are genotoxic [33]. An important enzyme involved in the 4-hydroxylation of estrogen is CYP1B1 [34]. After hydroxylation, these estrogens may further undergo sulfonation, glucuronidation (*UGT1A1*) or O-methylation (*COMT*), which increases the water solubility and therefore the excretion of these metabolites [35].

The *CYP1B1 *Val432Leu polymorphism has been associated with breast cancer in an Asian study [36] but not in two studies of Caucasians [36-38]. A Swedish case-control study observed an increased risk of breast cancer in Leu/Leu carriers in comparison with Val/Val carriers in women who had used HT for longer than 4 years [39]. However, a cross-sectional study showed no association between *CYP1B1 *genotype and mammographic density in women using HT [40]. In our study the *CYP1B1 *Val432Leu polymorphism predicted the change in MPD in women randomized to EPT, although this finding was no longer statistically significant after adjustment for multiple comparisons. There was no consistent dose effect with Leu alleles because heterozygotes had the lowest density increase. Our findings are therefore consistent with the available data and we cannot exclude the possibility that this gene might have a role in gene–environment interactions in EPT users.

The enzyme encoded by *COMT *is responsible for the conjugation and inactivation of catechol estrogen. A Val158Met polymorphism has been associated with lower activity of this enzyme [41] and is associated with increased plasma levels of 17β-estradiol in postmenopausal women taking ET [42]. In a recent cross-sectional study we reported a statistically significant association between the Met/Met allele and MPD in current users of HT (ET) [43]. In the present study, women assigned to ET in the EPAT study who possessed this high-risk variant showed a statistically significant increase in MPD compared with Val/Val homozygotes, but this effect was not observed in the ET arm of WELL-HART, in which the participants all had angiographically demonstrable coronary artery disease. We found no evidence that the *UGT1A1 *polymorphism was associated with MPD increase in women assigned to ET or EPT.

Strengths of our study included the randomized design, the use of a validated method and an experienced reader to assess mammographic density. However, there were several limitations. Study subjects represented only 57% and 47% of those originally randomized to EPAT and WELL-HART, respectively, because several of the participants in the parent trials had died or were lost to follow-up after the completion of the original trial. The small sample size, particularly in the EPT arm, limited our power to detect gene–environment interactions. However, it is unlikely that this could have biased our results and caused the apparent associations between genotype and change in MPD with treatment, because the most likely effect of this loss to follow-up would be to obscure a true association by a loss of statistical power. It is possible that some or all of the associations observed represent chance findings (false positives) due to multiple testing; however, our main findings of the effect of HT on MPD and the interaction with *AKR1C4 *genotype are statistically significant after conservative correction with Bonferroni's technique.

Another limitation is that women assigned to EPT and a subset of those on ET were drawn from a select study population with diagnosed cardiovascular disease and poor general health (the WELL-HART study). It is therefore unclear to what extent our findings can be generalized to populations with better health. The fact that we found that the *COMT *polymorphism modified the effect of ET on MPD in the EPAT study but not in the WELL-HART study suggests that the women with angiographically detected heart disease in the WELL-HART study might have been different. However, similar MPD changes were observed in the ET arms of both the EPAT and the WELL-HART study. Further, our finding of the magnitude of the increase in MPD associated with EPT use was similar to that recently reported from a trial of EPT use [11]. Thus, although we cannot exclude the possibility that the observed modifying effects of the *AKR1C4 *genotype were due to some other characteristic among these women with angiographically detectable heart disease, we find it unlikely.

## Conclusion

This is the first study to investigate genetic determinants of MPD changes in women randomized to ET, EPT or placebo. Although plausible, it is still unknown whether women with the greatest increase in MPD in response to EPT are at higher risk for breast cancer associated with EPT use than other women. Much research in this area remains to be done, but our findings from this pilot study suggest that the magnitude of the increase in MPD might be greater in women with a genetically determined lower activity of some enzymes that metabolize EPT.

## Abbreviations

AKR1C4 = aldo-keto reductase 1C4; BMI = body mass index; COMT = catechol-O-methyltransferase; CYP1B1 = cytochrome P450 1B1; EPAT = Estrogen in the Prevention of Atherosclerosis Trial; EPT = estrogen and progestin therapy; ET = estrogen therapy; HT = hormone therapy; MPD = mammographic percentage density; UGT1A1 = UDP-glucuronosyltransferase 1A1; WELL-HART = Women's Estrogen–Progestin Lipid-Lowering Hormone Atherosclerosis Regression Trial.

## Competing interests

The author(s) declare that they have no competing interests.

## Authors' contributions

SJL participated in the study design, data collection, conducted the statistical analyses and drafted the manuscript. WJM and HNH designed the original clinical trials and participated in the design of the study. DVB and WW performed the genetic analyses. SAI and CAH assisted in setting up the genetic analyses. YRP participated in study design and was responsible for obtaining the mammograms of study participants. MCP participated in the study design and drafting of the manuscript. GU conceived the study, participated in its design and coordination and helped to draft the manuscript. All authors read and approved the final manuscript.

## Figures and Tables

**Table 1:** Baseline characteristics by study and treatment group in women from the EPAT and WELL-HART trials

Characteristic	EPAT (*n *= 127)			WELL-HART (*n *= 105)				
	Placebo (*n *= 57)	ET (*n *= 70)	*P*	Placebo (*n *= 37)	ET (*n *= 34)	EPT (*n *= 34)	P*	*P*^†^
Age at baseline mammogram, years (mean ± SEM)	62.3 ± 1.0	60.2 ± 0.8	0.10	64.7 ± 1.0	61.8 ± 1.2	64.1 ± 1.1	0.15	0.005
Years since menopause at baseline (mean ± SEM)	15.1 ± 1.4	12.5 ± 1.0	0.12	18.6 ± 1.6	15.9 ± 1.4	19.5 ± 1.9	0.28	0.001
Number of deliveries (live and stillbirths) (mean ± SEM)	2.4 ± 0.2	2.3 ± 0.2	0.75	4.5 ± 0.6	3.6 ± 0.4	4.1 ± 0.5	0.47	0.0001
Race, *n *(%)								
White, non-latina	34 (59.7)	40 (57.1)		12 (32.4)	7 (20.6)	14 (41.2)		
Black, non-latina	6 (10.5)	10 (14.3)		4 (10.8)	10 (29.4)	5 (14.7)		
Latina	10 (17.5)	13 (18.6)		20 (54.1)	9 (26.5)	11 (32.4)		
Asian or Pacific Islander	7 (12.3)	7 (10.0)	0.91	1 (2.7)	8 (23.5)	4 (11.8)	0.01^‡^	0.0005
Education, *n *(%)								
High school graduate or less	8 (14.0)	9 (12.9)		20 (54.1)	15 (44.1)	15 (44.1)		
Trade or business school/some college	29 (50.9)	33 (48.6)		11 (29.7)	12 (35.3)	13 (38.2)		
Bachelor's degree or more	20 (35.1)	27 (38.6)	0.92	6 (16.2)	7 (20.6)	6 (17.7)	0.90	0.0001
Family history of breast cancer (first-degree relative), *n *(%)	9 (15.8)	6 (8.6)	0.21	2 (5.4)	7 (20.6)	3 (8.8)	0.14^‡^	0.91
Age at menarche, years (mean ± SEM)	12.7 ± 0.2	12.6 ± 0.2	0.86	12.7 ± 0.3	12.8 ± 0.3	13.3 ± 0.3	0.32	0.21
Ever used postmenopausal hormones, *n *(%)	36 (63.2)	42 (60.0)	0.72	13 (35.1)	15 (44.1)	21 (61.8)	0.08	0.02
BMI, kg/m^2 ^(mean ± SEM)	28.7 ± 0.7	28.7 ± 0.7	0.96	30.8 ± 1.0	31.4 ± 1.0	30.2 ± 1.1	0.73	0.007
Mammographic density at baseline, % (mean ± SEM)	17.8 ± 2.3	22.3 ± 1.9	0.13	7.9 ± 1.8	10.8 ± 2.1	13.3 ± 2.3	0.19	0.0001

*Comparison of characteristics by treatment group, χ^2 ^test for comparison of categorical variables, analysis of variance for comparison of means; ^†^comparison of characteristics between trials (EPAT versus WELL-HART), χ^2 ^test for comparison of categorical variables, analysis of variance for comparison of means; ^‡^Fisher's exact test. EPAT, Estrogen in the Prevention of Atherosclerosis Trial; ET, 1 mg/day micronized 17β-estradiol; EPT, 1 mg/day micronized 17β-estradiol with 5 mg/day medroxyprogesterone acetate for days 19 to 30 each month; WELL-HART, Women's Estrogen–Progestin Lipid-Lowering Hormone Atherosclerosis Regression Trial.

**Table 2:** Change in mammographic percentage density by treatment assignment

Treatment	Change in mammographic density (%)			
	Unadjusted mean ± SEM	*P*	Adjusted mean ± SEM*	*P*
EPAT				
Placebo (*n *= 57)	- 0.7 ± 0.8	Ref.	- 0.9 ± 0.9	Ref.
ET (*n *= 70)	3.4 ± 0.7	0.003	4.0 ± 0.8	0.0001
WELL-HART				
Placebo (*n *= 37)	0.8 ± 1.4	Ref.	0.06 ± 1.7	Ref.
ET (*n *= 34)	4.8 ± 1.5	0.06	5.6 ± 1.5	0.02
EPT (*n *= 34)	7.8 ± 1.5	0.001	7.8 ± 1.6	0.0005
*P *homogeneity of treatment effect between trials				0.76
All women				
Placebo (*n *= 95)	- 0.02 ± 0.8	Ref.	0.02 ± 0.8	Ref.
ET (*n *= 104)	3.9 ± 0.7	0.0003	4.6 ± 0.8	0.0001
EPT (*n *= 34)	7.8 ± 1.3	0.0001	7.2 ± 1.5	0.0001

*Adjusted for mammographic percentage density at baseline, race, age at baseline, years since menopause, past use of hormone therapy, body mass index (BMI) at baseline, change in BMI on trial and study group. EPAT, Estrogen in the Prevention of Atherosclerosis Trial; EPT, estrogen and progestin therapy; ET, estrogen therapy; WELL-HART, Women's Estrogen–Progestin Lipid-Lowering Hormone Atherosclerosis Regression Trial.

**Table 3:** Least-square mean change in mammographic percentage density by genotype and treatment arm, adjusted

Genotype	Placebo			ET			EPT			
	Mean (*N*)	SEM	P*	Mean (*N*)	SEM	*P*	Mean (*N*)	SEM	*P*	*P*^†^
*COMT *Val158Met										
Val/Val	- 0.1 (30)	1.4	Ref.	3.7 (30)	1.4	Ref.	8.0 (12)	2.3	Ref.	
Val/Met	0.5 (45)	1.2	0.74	4.3 (48)	1.1	0.72	6.2 (19)	1.9	0.54	
Met/Met	- 0.6 (19)	1.8	0.82	6.0 (25)	1.6	0.27	9.8 (3)	4.5	0.72	0.77
*CYP1B1 *Val432Leu										
Val/Val	- 0.7 (15)	1.9	Ref.	4.7 (23)	1.6	Ref.	6.9 (7)	2.9	Ref.	
Leu/Val	- 0.01 (42)	1.2	0.75	5.8 (44)	1.2	0.57	1.3 (13)	2.1	0.11	
Leu/Leu	0.9 (36)	1.2	0.47	3.6 (37)	1.2	0.61	14.5 (13)	2.1	0.03	0.0004
*UGT1A1*										
<7/<7 TA repeats	0.3 (37)	1.3	Ref.	5.7 (50)	1.1	Ref.	7.1 (18)	1.9	Ref.	
<7/≥ 7 TA repeats	- 0.5 (43)	1.2	0.60	3.3 (39)	1.3	0.15	8.7 (12)	2.3	0.57	
≥ 7/≥ 7 TA repeats	0.6 (14)	2.1	0.92	4.4 (14)	2.1	0.58	5.4(3)	4.6	0.74	0.77
*AKR1C4 *L311V										
Leu/Leu	- 0.3 (72)	0.9	Ref.	4.3 (79)	0.8	Ref.	4.0 (25)	1.6	Ref.	
Leu/Val	1.5 (21)	1.6	0.30	5.5 (25)	1.5	0.47	16.7 (7)	2.8	0.0001	
Val/Val	0.6 (1)		0.90	(0)			29.3 (1)		0.0007	0.001

Figures are adjusted for race, age at baseline, years since menopause, body mass index (BMI) at baseline, change in BMI on trial, mammographic percentage density at baseline, past use of hormone therapy and study group.

*Analysis of covariance *P *for comparison of means; ^†^analysis of covariance *P *for ET/EPT × genotype interaction. *AKR1C4*, aldo-keto reductase 1C4; *COMT*, catechol-O-methyltransferase; *CYP1B1*, cytochrome P450 1B1; EPT, estrogen and progestin therapy; ET, estrogen therapy; *UGT1A1*, UDP-glucuronosyltransferase 1A1.
